# Overexpression of Activating Transcription Factor 3 Alleviates Cardiac Microvascular Ischemia/Reperfusion Injury in Rats

**DOI:** 10.3389/fphar.2021.598959

**Published:** 2021-02-19

**Authors:** Yi Liu, Yisen Hu, Jingjie Xiong, Xiaocong Zeng

**Affiliations:** ^1^ Department of Cardiology, The First Affiliated Hospital of Guangxi Medical University, Nanning, China; ^2^ Guangxi Key Laboratory Base of Precision Medicine in Cardio-Cerebrovascular Diseases Control and Prevention, Nanning, China; ^3^ Guangxi Clinical Research Center for Cardio-Cerebrovascular Diseases, Nanning, China; ^4^ School of Basic Medical Sciences, Guangxi Medical University, Nanning, China

**Keywords:** Ischemia/reperfusion, microvascular injury, activating transcription factor 3, toll-like receptor 4, inflammatory response, oxidative stress

## Abstract

Activating transcription factor 3 (ATF3) has been confirmed to be responsive to oxidative stress and to negatively regulate the activity of Toll-like receptor 4 (TLR4). However, the effect of ATF3 on cardiac microvascular ischemia/reperfusion (I/R) injury remains unknown. The GEO2R online tool was employed to obtain differentially expressed genes GSE4105 and GSE122020, in two rat I/R injury microarray datasets. We established a rat myocardial I/R model *in vivo*, and also generated an *in vitro* hypoxia/reoxygenation (H/R) model of cardiomyoblast H9c2 cells**.** Overexpression of ATF3 was achieved by adenoviral-mediated gene transfer (Ad-ATF3). Rats were randomly divided into four groups: sham, I/R, I/R + Ad-Lacz (as a control), and I/R + Ad-ATF3. ELISA, CCK-8, DCFH-DA probe, qRT-PCR and Western blotting were used to determine the expression of ATF3, oxidative indices, cellular injury and TLR4/NF-κB pathway-associated proteins. Transmission electron microscopy, immunohistochemistry and immunofluorescence were used to detect the leukocyte infiltration and the alteration of microvascular morphology and function *in vivo*. Echocardiographic and hemodynamic data were also obtained. Bioinformatics analysis revealed that ATF3 was upregulated in I/R myocardia in two independent rat myocardial I/R models. Cardiac microvascular I/R injury included leukocyte infiltration, microvascular integrity disruption, and microvascular perfusion defect, which eventually resulted in the deterioration of hemodynamic parameters and heart function. Ad-ATF3 significantly restored microvascular function, increased cardiac microvascular perfusion, and improved hemodynamic parameters and heart function. Mechanistically, Ad-ATF3 ameliorated oxidative stress, inhibited TLR4/NF-κB pathway activation and down-regulated the expression of downstream proinflammatory cytokines in I/R myocardium *in vivo* and in H/R H9c2 cells *in vitro*. ATF3 overexpression protects against cardiac microvascular I/R injury in part by inhibiting the TLR4/NF-κB pathway and oxidative stress.

## Introduction

Primary percutaneous coronary intervention is the standard treatment for reducing myocardial necrosis and improving clinical prognosis in patients undergoing acute ST-segment elevation myocardial infarction (Ibanez et al., 2018). However, unfortunately, a microvascular reperfusion injury can occur while epicardial blood flow is being restored by reperfusion therapy. This event is known as the “no-reflow (NR)” phenomenon (Allencherril et al., 2019) and is regarded as myocardial tissue hypoperfusion. The underlying pathophysiology of this event involves cardiac microvascular ischemia/reperfusion (I/R) injury. Recent studies indicate that the “no-reflow” phenomenon or cardiac microvascular I/R injury can be primarily attributed to cardiac microvascular damage. The common presentation of microvascular damage includes severe endothelial cell swelling, rupture of the microvessel wall and hemorrhage into the interstitial space after reperfusion (Kloner et al., 1974; Heusch, 2019). It is well established that oxidative stress is a primary culprit of I/R injury (Yu et al., 2016; Li et al., 2018). I/R can lead to a “burst” of reactive oxygen species (ROS) that are released from mitochondria (Cadenas, 2018). Recent studies have demonstrated that oxidative stress is an influential mechanism of endothelial damage, cardiac microcirculation endothelial cell (CMEC) death and microvascular I/R injury (Zhou et al., 2018d; Zhou et al., 2019). At the molecular and cellular level, the activation of inflammatory signaling pathways, leucocyte adherence and microembolization results in microvascular endothelial dysfunction and causes microvascular endothelial hyperpermeability and junctional loss, leading to microcirculatory dysfunction (Oikonomou et al., 2018; Yu et al., 2019). The restoration of blood flow to the ischemic vascular bed induces microvascular damage through multiple mechanisms. Immediately after reperfusion, the extensive microcirculatory dysfunction results in the insufficient availability of blood, energy, oxygen and nutrients to the cardiomyocytes. Importantly, this occurs even though the normal epicardial flow is restored and results in the further exacerbation of myocardial damage (Zhou et al., 2018d; Wang et al., 2020). Therefore, improving microvascular perfusion is considered an efficient therapeutic method to alleviate or abolish the NR phenomenon, and effectively protects the myocardium from I/R injury (O’farrell et al., 2017; Ozawa et al., 2018).

Toll-like receptors (TLRs), members of the transmembrane protein family belonging to pattern recognition receptors (PRRs), induce inflammation by conveying extracellular antigen signaling into intracellular signaling cascades (Kawai and Akira, 2007). Among the TLR family members, Toll-like receptor 4 (TLR4) has been shown to play an important role in the induction of systematic inflammation and activation of the downstream NF-κB pathway during myocardial I/R (Zhang et al., 2017). The activation of this pathway can further promote the expression of proinflammatory cytokines and aggravate myocardial injury (Dong et al., 2018). Conversely, the suppression of TLR4/NF-κB-mediated inflammatory responses protect rats against myocardial I/R injury (Yuan et al., 2018).

Activating transcription factor-3 (ATF3) belongs to the ATF/cyclic adenosine mono-phosphate response element binding (CREB) family of basic leucine zipper TFs (Brooks et al., 2015). Appropriate ATF3 expression is essential for maintaining normal cellular function, whereas abnormal expression thereof is involved in a variety of pathophysiological processes (Hai et al., 2010). ATF3 is considered an adaptive response gene and functions as an activator and repressor of transcription (Lu et al., 2006). ATF3 is implicated in various physiological and pathological processes throughout the cardiovascular system, including the inflammatory response, oxidative stress, apoptosis, endoplasmic reticulum stress and cardiac remodeling (Kalfon et al., 2017; Li et al., 2017; Zhou et al., 2018b). ATF3 has been confirmed to be responsive to ROS-mediated oxidative stress (Zhou et al., 2018b; Lin and Cheng, 2018). Previous studies have demonstrated that the ATF3 gene is induced when organs suffer from oxidative stress due to I/R (Yin et al., 1997; Allen-Jennings et al., 2001). Furthermore, ATF3 overexpression can inhibit oxidative stress-induced cell death in HK2 cells (Yoshida et al., 2008). On the other hand, a recent study confirmed that the expression of ATF3 is upregulated by TLR4/NF-κB pathway activation (De Nardo, 2015). Studies have shown that ATF3 serves as a part of negative feedback loop to modulate the TLR4-stimulated expression of proinflammatory genes (Gilchrist et al., 2006; Whitmore et al., 2007; Boespflug et al., 2014). A recent study showed that the genetic deletion of ATF3 abolished the cardioprotective effects of ischemic preconditioning in mouse hearts subjected to ischemia-reperfusion injury (Brooks et al., 2014). However, genetic deletion of ATF3 did not influence leukocyte recruitment in mouse hearts undergoing ischemic preconditioning alone (Brooks et al., 2014). These findings suggest the possibility that ATF3 inhibits oxidative stress and the TLR4/NF-κB-mediated inflammatory response during myocardial ischemia/reperfusion.

We hypothesize that the overexpression of ATF3 may relieve the inflammatory response and reduce microvascular permeability by negatively regulating the activity of TLR4/NF-κB signaling. This could in turn alleviate oxidative stress, ameliorate microvascular damage and eventually attenuate cardiac microvascular I/R injury. To verify this hypothesis, we first established an *in vivo* rat myocardial I/R model and an *in vitro* hypoxia/reoxygenation (H/R) model using H9C2 cells, a rat cardiomyoblast cell line (Watkins et al., 2011). We then examined the effects of overexpressing ATF3 on the inflammatory response, leukocyte infiltration, oxidative stress, microvascular permeability, microvascular perfusion and cardiac function in I/R rats. Our findings suggested that ATF3 protected hearts against cardiac microvascular I/R injury and may serve as a novel therapeutic target in the clinic**.**

## Materials and Methods

### Screening of Differentially Expressed Genes (DEGs)

Two gene expression datasets obtained from the rat myocardial I/R models, GEO: GSE4105 and GSE122020, were identified from the GEO database. GSE4105 included the gene expression profile of rats undergoing surgery for left anterior descending coronary artery (LAD) ligation. LAD lasted for 30 min and was followed by reperfusion. Ventricular tissue samples were obtained 2 and 7 days after reperfusion. The candidate DEGs at 2 days after I/R (n = 3) and 2 days after a sham procedure (n = 3) were screened using GEO2R. The threshold was set as *p* < 0.05 and the log fold change >2. GSE122020 included the gene expression data of three groups, the sham, I/R, and I/R + remote ischemic postconditioning groups. The rat I/R model was established by performing LAD ligation for 45 min followed by reperfusion for 24 h. Myocardial tissues were obtained at the endpoint of reperfusion. The candidate DEGs between I/R (n = 3) and sham (n = 3) were screened using GEO2R and the threshold was also set as *p* < 0.05 and the log fold change >2. DEGs obtained from these two datasets were analyzed using the R software and were plotted as a heatmap. The overlapping DEGs of these two datasets were further analyzed using the online Venny Diagram tool.

### Adenoviral Constructs

Recombinant adenovirus containing ATF3 cDNA (Ad-ATF3) was provided by Shanghai Genechem Company (Shanghai, China). The sequences of the ATF3 primers used for this subcloning were as follows: forward 5′-AAA​AAG​CTT​ATG​ATG​CTT​CAA​CAT​CCA​GG-3′ and reverse 5′-TTT​GAA​TTC​TTA​GCT​CTG​CAA​TGT​TCC​TT-3’. Adenoviruses expressing the escherichia coliβ-galactosidase (LacZ) gene (Ad-LacZ) were used as a control. Viruses were amplified and titrated in HEK293 cells according to manufacturer’s instruction.

### Rat I/R Model and Adenoviral Gene Transfer *in vivo*

**FIGURE 1:**
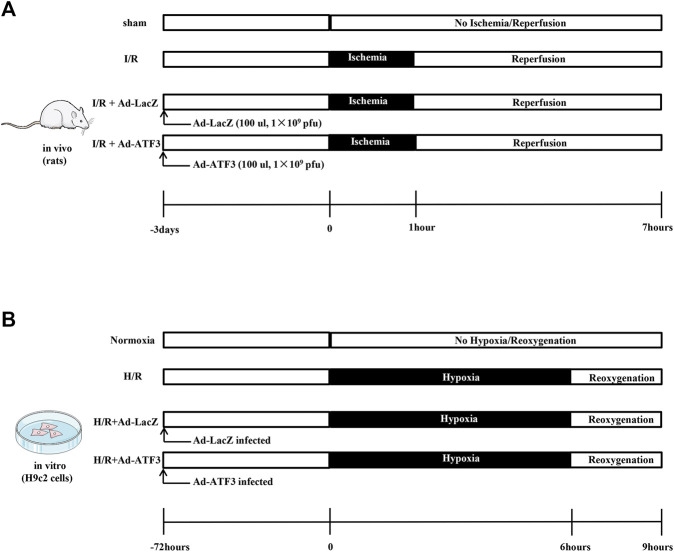
Schematic illustration of experimental protocols **(A)** Recombinant adenovirus (100 ul, 1 × 10^9^ pfu) was delivered directly into the ventricle via intramyocardial injections. Three days later, rats were subjected to 1 h of myocardial ischemia and 6 h of reperfusion **(B)** H9c2 cells were infected with Ad-ATF3 or Ad-LacZ. After 72 h, H9c2 cells were subjected to 6 h of hypoxia followed by 3 h of reoxygenation to induce an H/R injury.

The animal experiments performed in this study were implemented strictly in compliance with the principle of care and use of laboratory animals and met the standards of the Animal Welfare and Ethics Committee in Guangxi Medical University. Thirty-two Sprague-Dawley (SD) male rats (200–250 g) were randomly divided into four groups: sham, I/R, I/R + Ad-LacZ, and I/R + Ad-ATF3. Three days before the surgical occlusion, recombinant adenovirus (100 ul, 1 × 10^9^ pfu) was delivered directly into the peripheral regions of the infarct area at the anterior and lateral aspects of the ventricle via intramyocardial injections using a precision syringe (Tenhunen et al., 2006; Hong et al., 2014). Rats were anesthetized with an intraperitoneal injection of 3% sodium pentobarbital (30 mg/kg). The LAD was then ligated for 1 h to induce myocardial ischemia and was followed by the release of the ligation for 6 h to simulate reperfusion (Figure 1A). By the end of reperfusion, venous blood was collected and serum samples were separated by centrifugation. Evans blue and triphenyltetrazolium chloride (TTC) staining were performed to examine the infarct area (IA) and calculate the area at risk (AAR). All rats were sacrificed by cervical dislocation and heart tissues were obtained for further histological and biochemical examination.

### Cell Transfection and Hypoxia/Reoxygenation (H/R) Protocol

The H9c2 cardiomyblast cell line was obtained from the Cell Bank of the Chinese Academy of Sciences (Shanghai, China) and cultured in Dulbecco’s modified Eagle’s medium (DMEM) containing 10% fetal bovine serum (FBS) at 37°C in 95% air and 5% CO_2_. After 36 h of culture, H9c2 cells were infected with Ad-ATF3 or Ad-LacZ. To overexpress or knock down TLR4, cells were transfected with Ad-TLR4 (Shanghai GeneChem Co., Ltd., Shanghai, China) or Si-TLR4 (sc-156001, Santa Cruz Biotechnology, Inc., Santa Cruz, CA, USA). H/R was induced at 72 h after transfection. Briefly, H9c2 cells were incubated in an anaerobic environment (serum- and glucose-free medium) within the anoxic tank (Thermo Fisher Scientific, Waltham, MA, USA) with 1% O_2_, 5% CO_2_, and 94% N_2_ for 6 h at 37°C, followed by a transfer to a normoxic condition with a normal medium for reoxygenation (95% air and 5% CO_2_) for 3 h (Figure 1B).

### Hemodynamic Measurement and Echocardiography

The right common carotid artery was cannulated and connected to the MPA cardiac function system (Shanghai Alcott Biotech Co. Ltd., China) for continuous hemodynamic monitoring. At the end of reperfusion, hemodynamic parameters, including left ventricular end-diastolic pressure (LVEDP), left ventricular systolic pressure (LVSP), +dp/dpmax, and -dp/dtmax were recorded as an average of ten cardiac cycles. A Philips sonos7500 ultrasound system (Philips Healthcare, Amsterdam, The Netherlands) was used to measure left ventricular fractional shortening (LVFS) and ejection fraction (EF).

### Enzyme-Linked Immunosorbent Assay (ELISAs)

ELISA kits (Dakewe Biotech, Shenzhen, China) were used to determine the contents of TNF-α, IL-1β, and IL-6 in the supernatants of cells and rat serum. The levels of CK-MB and cardiac troponin T (cTnT) in rat serum were also determined using ELISA kits (cat. no. orb410928 and orb442655, Biorbyt, Cambridge, United Kingdom).

### Assay of Oxidative Stress and Measurement of Cellular Injury

The intracellular ROS generation was measured with a DCFH-DA (2,7-dichlorofluorescein diacetate) probe according to the instructions of the ROS kits (Nanjing Jiancheng Biological Product, Nanjing, China). Briefly, H9c2 cells were incubated with 10 μmol DCFH-DA at 37°C for 30 min in the dark. The fluorescence intensity was measured using a Fluorospectrophotometer with 488 nm excitation and 525 nm emission filters. The level of methane dicarboxylic aldehyde (MDA), the activity of superoxide dismutase (SOD) and glutathione peroxidase (GSH-Px) in H9c2 cell homogenates were determined using an ELISA kit according to the manufacturer’s instructions (Shanghai Enzyme-Linked Biotechnology Co. Ltd., Shanghai, China). The level of ROS,MDA, SOD and GSH-Px in rat serum was also determined using ELISA kits (Wuhan Colorful Gene Biological Technology Co., Ltd., Wuhan, China). In accordance with the manufacturers’ instructions, cell viability and lactate dehydrogenase (LDH) release were determined using a cell counting kit-8 (CCK-8) and LDH assay kit (Nanjing Jiancheng Bioengineering Institute, China), respectively, to assess the cellular injury in H9C2 cells.

### Transmission Electron Microscopy (TEM)

The ultrastructural changes of capillaries in the hearts subjected to I/R injury were identified by transmission TEM (HT7800, Tokyo, Japan). Myocardial samples for TEM examination were prepared using the same methods as previously described (Zhong et al., 2007). The ImageJ 1.48 software (NIH, MD, USA) was used to quantify the area of the capillary lumen and the area occupied by endothelial cells based on previously established methods (Marszalek et al., 2000). Endothelial cell junctions, including junction length, were also examined by TEM (Hakanpaa et al., 2018) and measured using the ImageJ 1.48 software. The cortical protein complex area typically appeared as electron-dense when viewed under TEM. The ratio of electron density relative to the overall length of cell contact was also calculated.

### Gelatin ink Staining

Microvascular perfusion was examined by gelatin-ink staining according to a previously detailed method (Zhou et al., 2018d). Briefly, the gelatin-ink (3% gelatin and ink) was administered intravenously through the jugular vein after reperfusion. Rat hearts were removed and stored at 4°C for 1 h. Myocardial tissue tissues were fixed with 4% paraformaldehyde and then frozen sections were prepared. The gelatin-ink intensity images were taken at ×200 magnification and quantified with the ImageJ 1.48 software.

### Histopathology, Immunohistochemistry, and Immunofluorescence

Paraformaldehyde-fixed myocardial tissues were treated with gradient alcohol dehydration and then embedded in paraffin. Hematoxylin and eosin (HE) and immunohistochemistry staining was implemented in 4 µm thick sections of heart tissues. Immunofluorescence staining, fixation and permeabilization were performed according to routine procedures. Tissue sections were blocked in 5% normal goat serum for 1 h at room temperature and then incubated with primary antibodies of interest at 4°C overnight. The primary antibodies used for immunohistochemistry and immunofluorescence staining are as follows: VE-cadherin (1:100, Bioss, bs-0878R), F4/80 (1:100, Bioss, bs-11182R), CD31 (1:100, Abcam, ab24590), VCAM-1 (1:500, Abcam, ab134047), ICAM-1 (1:500, Abcam, ab171123), cTnT (1:400, Abcam, ab45932), and plasma Albumin (1:1,000, Abcam, ab8940). Nuclei were counter-stained with DAPI. At least five fields per rat with a high-density area in each group were randomly scored with the case Viewer Software (3D HISTECH) using a ×40 objective.

### Reverse Transcription (RT) and Real-Time Quantitative PCR (qPCR)

The TRIzol® reagent (Thermo Fisher Scientific, Pittsburgh, PA, USA) was used to isolate total RNA from H9c2 cells and cardiac tissues. RQ1 RNase-free DNase (Promega Corporation, Madison, WI, USA) was used to remove the contaminated genomic DNA from the total RNA. A PrimerScript RT reagent kit (Takara Bio Inc., Shiga, Japan) was used to reverse transcribe the RNA into cDNA, which was then subjected to qPCR using the SYBR Green PCR Master Mix on the ABI 7500 Real time PCR system. The sequence of primers used in this study are shown in Table 1. The PCR conditions included an initial period at 95°C for 10 min for denaturation, 40 cycles at 95°C for 15 sec and 60°C for 1 min. GAPDH was used as the reference. The 2^−∆∆Ct^ method was used to determine the relative expression levels of target genes.

**TABLE 1:** Primer sequences for amplification.

Target genes	Forward primers	Reverse primers
ATF3	5′- CCA​GAA​CAA​GCA​CCT​TTG​CC -3′	5′- CGG​CAT​TCA​CAC​TCT​CCA​GT-3′
TLR4	5′-AGA​AAC​TGC​TCG​GTC​AGA​CG-3′	5′- GGG​CTA​AAC​TCT​GGA​TGG​GG-3′
TNF-α	5′- CCT​CTT​CTC​ATT​CCT​GCT​C-3′	5′- CTT​CTC​CTC​CTT​GTT​GGG-3′
IL-1β	5′- CCC​TGA​ACT​CAA​CTG​TGA​AAT​AGC​A -3′	5′- CCC​AAG​TCA​AGG​GCT​TGG​AA -3′
Il-6	5′- ATT​GTA​TGA​ACA​GCG​ATG​ATG​CAC -3′	5′- CCA​GGT​AGA​AAC​GGA​ACT​CCA​GA -3′
GADPH	5′- CAA​GTT​CAA​CGG​CAC​AGT​CA -3′	5′- CCC​CAT​TTG​ATG​TTA​GCG​GG-3′

### Western Blot Analysis

Total proteins were purified from H9c2 cells and myocardial tissues using 1% radioimmune precipitation assay (RIPA) lysis buffer (Beyotime, Jiangsu, China). Nuclear and cytosolic fractions were purified using NE-PER Nuclear and Cytoplasmic Extraction Reagents (Thermo Fisher Scientific, Waltham, MA, USA). Protein concentrations were determined using the Bicinchoninic Acid Protein Assay kit (Pierce Biotechnology, IL, USA). Equivalent amounts of proteins were resolved in 10% SDS-PAGE gels and transferred to nitrocellulose membranes. After blocking in 5% non-fat milk for 1 h at room temperature, the membranes were incubated with the following primary antibodies at 4°C overnight: ATF3 (1:1,000, Abcam, ab216569), TLR4 (1:300, Abcam, ab 217,274), *p*-IκBα (1:1,000 Cell Signaling Technology, 9,246), IκBα (1:1,000 Cell Signaling Technology, 4,814), NF-κB p65 (1:200, Abcam, ab16502), *p*-eNOS (1:1,000, Abcam, ab138458), eNOS (1:1,000, Abcam, ab76198), Lamin B1 (1:1,000, Abcam, ab16048), GADPH (1:1,000, Abcam, ab8245). After washing, the membranes were incubated with the HRP-conjugated secondary antibody for 1 h at room temperature. The specific protein bands were visualized by chemiluminescence (Thermo Fisher Scientific, Inc.) and the intensity of protein bands were quantified using the ImageJ 1.48 software. GAPDH and Lamin B were used as an internal reference for cytoplasmic and nuclear proteins, respectively.

### Statistical Analysis

Data are presented as the mean ± standard deviation (SD). A one-way analysis of variance (ANOVA) were performed to compare the data of multiple groups and a Tukey’s honest significant difference test was used to control for multiple comparisons. All statistical analyses were conducted using the GraphPad Prism 8 (GraphPad, San Diego, CA, USA), and *p* < 0.05 was considered statistically significant.

## Results

### Bioinformatics analysis

**FIGURE 2:**
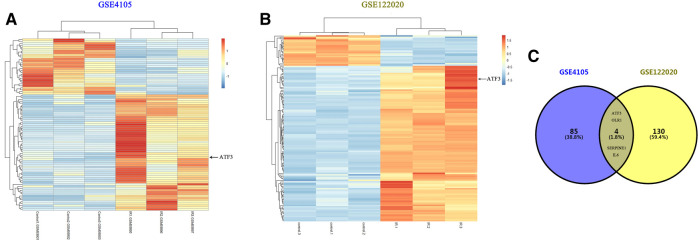
Bioinformatics analysis identified DEGs in two independent gene expression datasets related to myocardial I/R rat models **(A&B)** Heatmaps representing the screening results by bioinformatics analysis in two gene expression datasets (GSE4105 and GSE122020). Normalized gene expression values were used to represent the levels of DEGs in the I/R group compared with the control group. Each row represents a DEG, with each sample expression value being represented by one rectangle. The color gradation indicates the intensity of gene expression: red for the highest expression and blue for the lowest expression. ATF3 significantly increased in I/R-injured myocardium **(C)** Venn diagram showing the common DEGs in GSE4105 and GSE122020, including ATF3, OLR1, SERPINE and IL6.

We first performed online analysis using the GEO2R software to identify DEGs in two independent gene expression datasets, GEO: GSE4105 and GSE122020, which are related to the myocardial I/R rat models. For this analysis, we used a *p* value < 0.05 and |logFC|> 2 as cutoff criteria. A heatmap shows the identified DEGs between I/R and control (sham) groups in GSE4105 (Figure 2A) and GSE122020 (Figure 2B). Both heatmaps showed a significant increase in ATF3 expression after I/R, indicating that ATF3 may be potentially involved in the pathophysiology during myocardial I/R. Venn diagrams showed an intersection of the DEGs between GSE4105 and GSE122020 data subsets, which included four common genes with altered expression: ATF3, OLR1, SERPINE and IL6 (Figure 2C). Although it has been reported to be important for ischemic preconditioning (Brooks et al., 2014), the biological role of ATF3 in cardiac microvascular I/R has not been fully investigated. Therefore, in the present study, we focused on the potential roles of ATF3 in cardiac microvascular I/R injury.

### ATF3 Overexpression Inhibited TLR4/NF-κB Pathway Activation, Oxidative Stress and Cellular Injury in H/R

**FIGURE 3:**
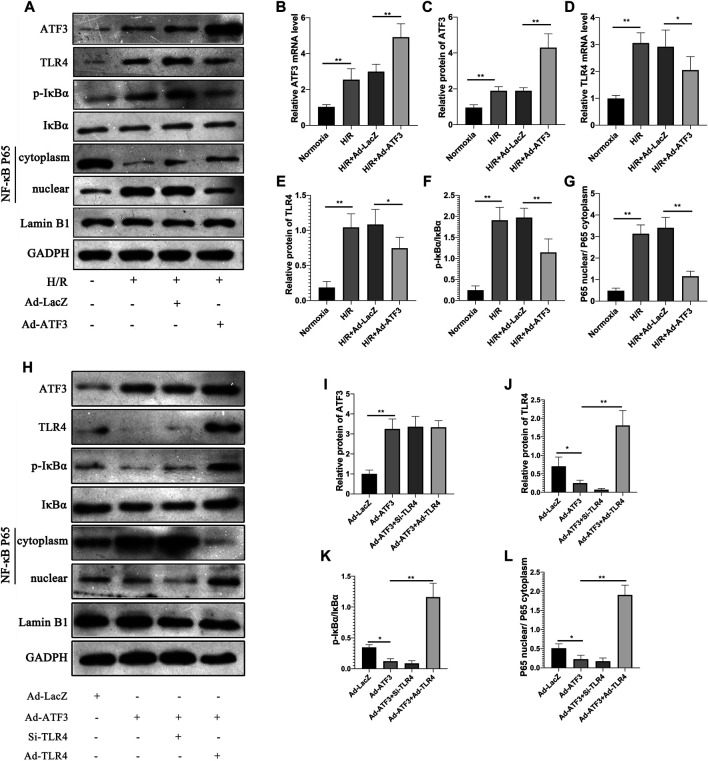
ATF3 overexpression inhibited TLR4/NF-κB pathway activation in H/R H9c2 cells **(A and H)** The expression level of ATF3, TLR4, and phosphorylation of IκBα, NF-κB p65 in the nuclear and cytoplasmic fractions were examined by Western blotting **(B and D)** The mRNA level of ATF3 and TLR4 in H9c2 cells were determined by qRT-PCR **(C, E-G, I-L)** Quantification of each protein band in **A and H** was performed with the ImageJ software. The intensity of cytoplasmic and nuclear proteins was normalized to GADPH and Lamin B, respectively. *p < 0.05, **p < 0.01. n = 6/group.

**FIGURE 4:**
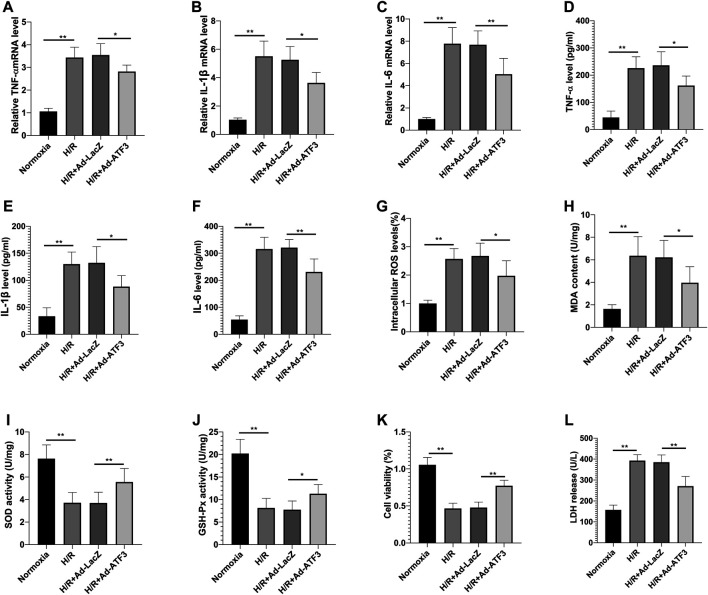
ATF3 overexpression inhibited H/R-induced inflammation, oxidative stress and cellular injury in H9c2 cells **(A–C)** The mRNA level of TNF-α, IL-1β and IL-6 in H9c2 cells was determined by qRT-PCR **(D–F)** The level of proinflammatory cytokines, TNF-α, IL-1β and IL-6 in H9c2 cells was determined using ELISA kits **(G–J)** The intracellular ROS generation was measured with a DCFH-DA probe, and the level of MDA, SOD and GSH-Px in H9c2 cells was determined using ELISA kits **(K,L)** Cell viability was detected by CCK-8. LDH was determined using a cytotoxicity assay kit. *p < 0.05, **p < 0.01. n = 6/group.

We first asked whether ATF3 could functionally interact with the TLR4/NF-κB pathway to mediate H/R injury. We overexpressed ATF3 through adenovirus-mediated transduction in H9c2 cells which were subjected to H/R. The success in adenoviral gene transduction was examined by RT-qPCR and Western blot analysis. As shown in Figures 3A–C, H/R stimulation increased ATF3 expression in H9c2 cells. However, Ad-ATF3 significantly increased ATF3 expression in H/R-stimulated H9c2 cells compared with the H/R + Ad-LacZ group at both the mRNA and protein levels. Also, H/R stimulation triggered TLR4 activation, which promoted IκBα phosphorylation and p65 nuclear translocation, thereby resulting in NF-κB activation (Figures 3A,D–G). The NF-kB activation in turn up-regulated the expression of downstream genes such as TNF-α, IL-1β, and IL-6 (Figures 4A–F). However, Ad-ATF3 significantly inhibited the activation of TLR4, IκBα phosphorylation and NF-κB p65 nuclear translocation in the H/R + Ad-ATF3 group compared with the H/R + Ad-LacZ group (Figures 3A,D–G). Accordingly, Ad-ATF3 down-regulated the expression of TNF-α, IL-1β and IL-6 at both the mRNA and protein levels in the H/R + Ad-ATF3 group (Figures 4A–F). To further verify and confirm the role of ATF3 in TLR4/NF-*κ*B pathway regulation (based on Ad-ATF3 transfected with H9c2 cells), we further transfected Ad-TLR4 (overexpression) or Si-TLR4 (knock down). The results showed that Ad-ATF3 significantly inhibited the expression of TLR4, IκBα phosphorylation and NF-κB p65 nuclear translocation. Interestingly, this effect was effectively reversed by co-transfection with Ad-TLR4 (Figures 3H–L). H/R up-regulated ROS production and MDA levels, but down-regulated the level of anti-oxidant factors such as SOD and GSH-Px. However, Ad-ATF3 induced the opposite effect as it decreased ROS and MDA levels and reversed the changes in SOD and GSH-Px expression (Figures 4G–J). In addition, H/R significantly reduced cell viability while increasing LDH release, both of which events were significantly reversed by ATF3 overexpression (Figures 4K,L). Taken together, ATF3 overexpression inhibits the H/R-induced TLR4/NF-κB pathway activation, inflammation, oxidative stress and cellular injury.

### ATF3 Overexpression Inhibited TLR4/NF-κB Pathway Activation and Oxidative Stress in I/R Rats

**FIGURE 5:**
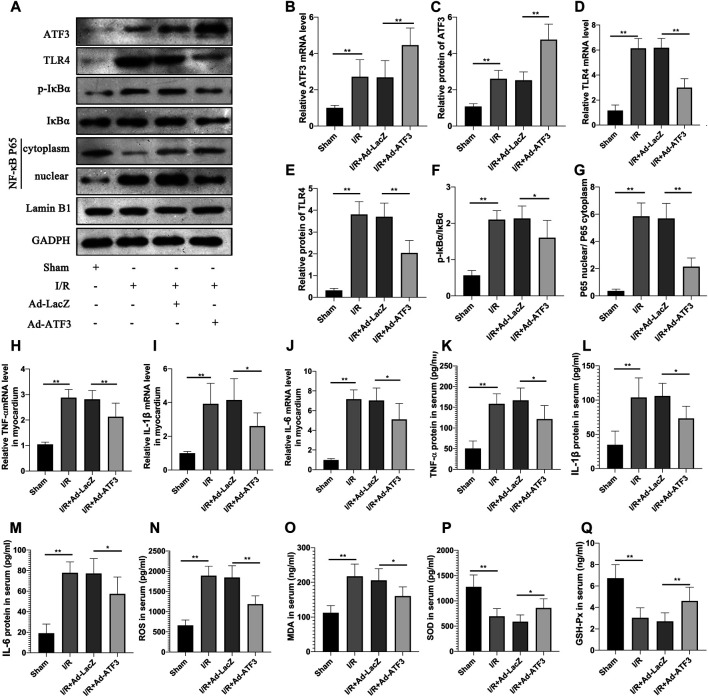
ATF3 overexpression inhibited TLR4/NF-κB pathway activation in I/R myocardium **(A)** The expression levels of ATF3, TLR4, phosphorylation of IκBα, NF- κB p65 in the nuclear and cytoplasmic fractions were examined by Western blotting **(B,D, H–J)** The mRNA level of ATF3, TLR4,TNF-α, IL-1β and IL-6 in myocardium were determined by qRT-PCR **(C,E–G)** Quantification of each protein band in A using the ImageJ software. The intensity of cytoplasmic and nuclear proteins were normalized to GADPH and Lamin B, respectively **(K–M)** The levels of serum proinflammatory cytokines, TNF-α, IL-1β and IL-6, were determined by ELISAs **(N–Q)** The level of serum ROS,MDA, SOD and GSH-Px were determined using ELISA kits. *p < 0.05, **p < 0.01. n = 8/group.

The above findings suggested that ATF3 overexpression suppressed the TLR4/NF-κB pathway activation and inflammation at the cellular level. We next evaluated the effects of ATF3 overexpression on TLR4/NF-κB pathway activity in a rat myocardial IR injury model *in vivo* using adenovirus-mediated ATF3 gene delivery via intra-myocardial injections. RT-qPCR and Western blotting were used to evaluate the success in this adenoviral-mediated gene transfer. As expected, myocardial I/R increased ATF3 expression in the myocardium. Ad-ATF3 significantly upregulated both mRNA and protein levels in myocardial I/R compared to the I/R + Ad-LacZ group (Figures 5A–C). As observed in H/R H9c2 cells, I/R significantly activated TLR4 activation, induced IκBα phosphorylation, and promoted NF-κB p65 nuclear translocation in the myocardium (Figures 5A,D–G). Accompanied by the TLR4/NF-κB pathway activation, the expression of its downstream genes, TNF-α, IL-1β and IL-6, were also substantially increased in the myocardium (Figures 5H–J) and serum (Figures 5K–M). Ad-ATF3 significantly inhibited TLR4 activation, IκBα phosphorylation and NF-κB p65 nuclear translocation compared with I/R + Ad-LacZ (Figures 5A,D–G). Accordingly, Ad-ATF3 down-regulated the levels of proinflammatory cytokines, TNF-α, IL-1β and IL-6, in both serum (Figures 5K–M) and the myocardium (Figures 5H–J). I/R injury increased the level of ROS and MDA, and decreased the level of SOD and GSH-Px in serum of rats. However, Ad-ATF3 induced the opposite effects by reducing ROS and MDA levels, and markedly upregulating SOD and GSH-Px expression (Figures 5N–Q). Thus, we argue that ATF3 overexpression inhibits the TLR4/NF-κB pathway activation inflammation and oxidative stress following myocardial I/R *in vivo*.

### ATF3 Overexpression Inhibited Leukocyte Infiltration in I/R Myocardium

**FIGURE 6:**
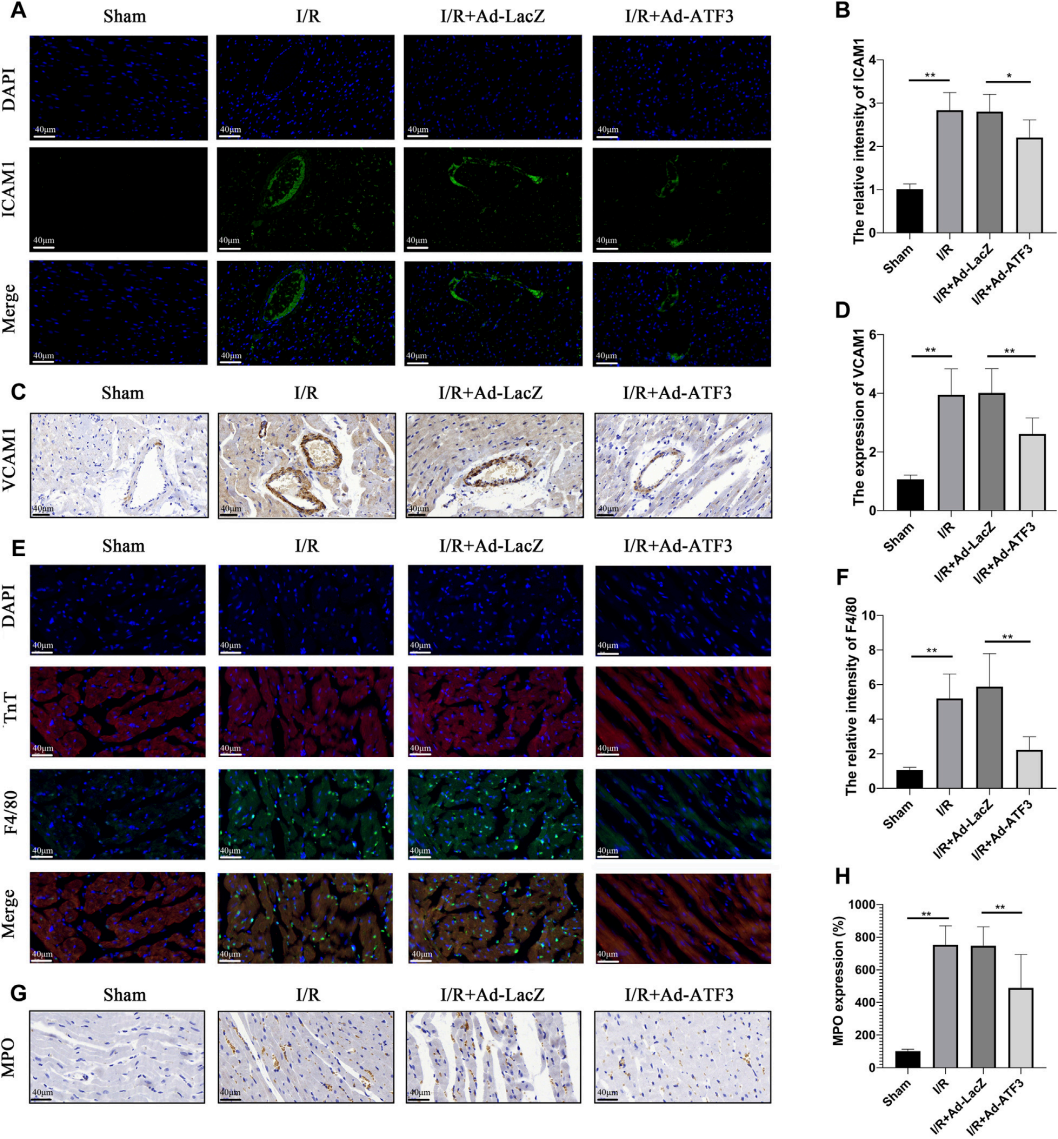
ATF3 overexpression inhibited leukocyte infiltration in I/R myocardium **(A and B)** ICAM-1 expression in the microvasculature was examined by immunofluorescence assay. At least 10 microvascular lumens in at least five randomly selected fields per group were observed **(C and D)** VCAM-1 expression in the microvasculature was examined by immunohistochemistry. At least 10 microvascular lumens in at least five randomly selected fields per group were observed **(E and F)** The neutrophils were stained with F4/80 and the myocardium was stained with cTnT using an immunofluorescence assay. Colocalization of F4/80 and cTnT represent neutrophils that migrated into the myocardium **(G and H)** Neutrophils were stained with MPO by immunohistochemistry. At least five randomly selected fields within the high-density area per group were observed. *p < 0.05, **p < 0.01.8/group.

We next examined whether ATF3 overexpression plays a role in regulating leukocyte infiltration. Myocardial I/R upregulated the levels of ICAM1 and VCAM1 on the microvascular surface (Figures 6A–D), which can aggravate leukocyte recruitment. Indeed, as shown in Figures 6E–H, more F4/80+ or MPO + leukocytes were present in the I/R myocardium. Compared to the I/R + Ad-LacZ group, the I/R + Ad-ATF3 group significantly down-regulated the levels of ICAM1 and VCAM1 on the microvascular surface and decreased the presence of F4/80+ or MPO + leukocytes in I/R myocardium.

### ATF3 Overexpression Improved Microvascular Integrity and Permeability in I/R Myocardium

**FIGURE 7:**
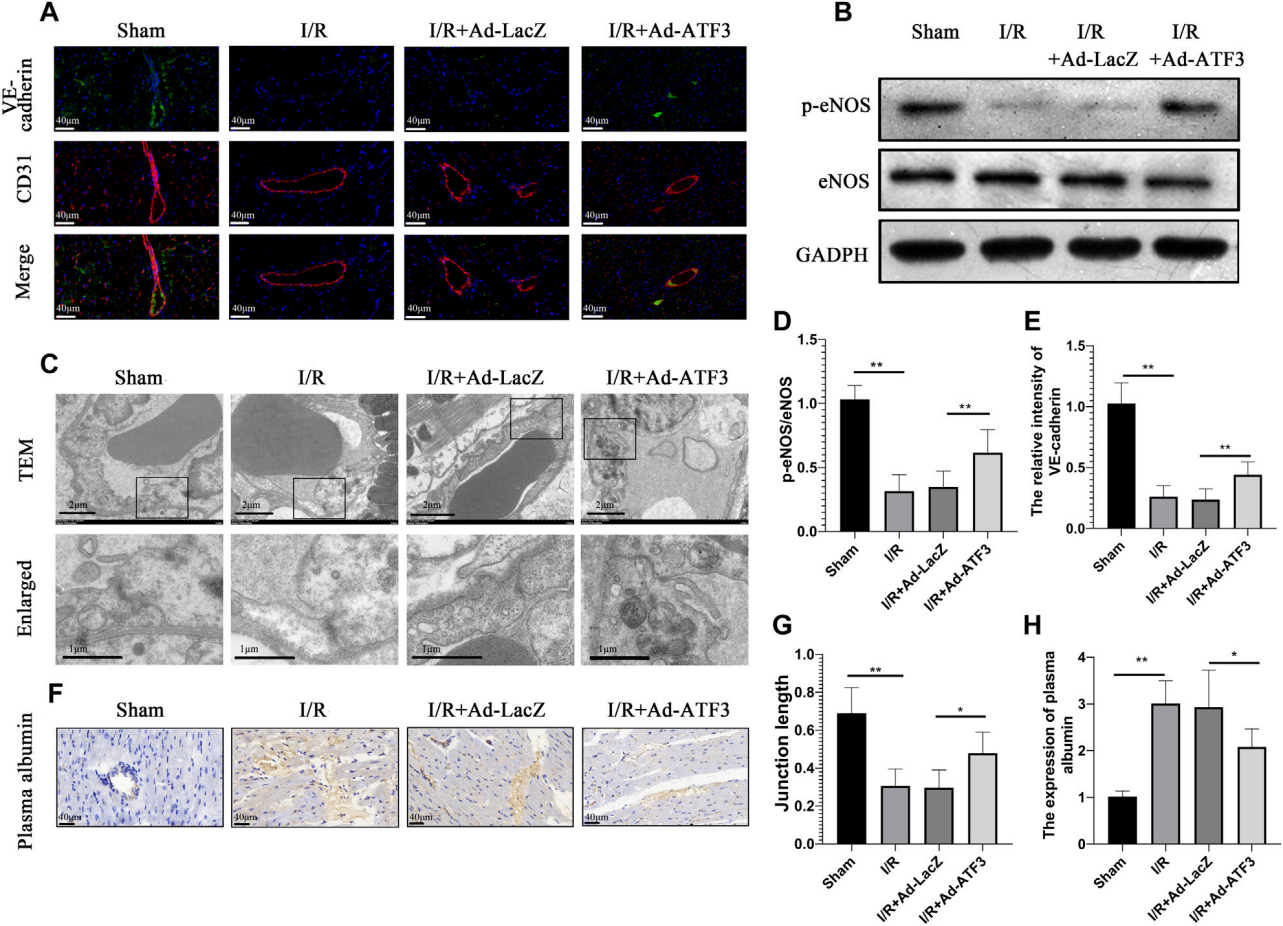
ATF3 overexpression maintained the microvascular barrier in I/R injury rats** (A and E)** Double immunofluorescence staining for VE-cadherin and CD31 was performed to detect the endothelial barrier integrity. IR caused the discontinuity of VE-cadherin expression as revealed by immunofluorescence, which was ameliorated by ATF3 overexpression **(B)** The expression levels of *p*-eNOS were determined by Western blots **(D)** Quantification of B by ImageJ software. The intensity of protein bands were normalized to GADPH **(C)** TEM reveals ultrastructure of endothelial adherent junctions in microvessels. Lower panels are the enlarged images of rectangular areas of the upper panels indicating the cellular contact and electron dense area (cortical protein complex) **(G)** The ratio of electron dense areas of intercellular junctions relative to the total length of intercellular contact **(F and H)** The plasma albumin was stained by immunohistochemistry to detected microvascular permeability. Plasma albumin leaked out from microvessels into myocardial tissues during I/R, which was attenuated by ATF3 overexpression. *p < 0.05, **p < 0.01. n = 8/group.

Previous studies have shown that inflammation and leukocyte infiltration further impaired microvascular integrity and permeability (Zhou et al., 2018b; Hakanpaa et al., 2018). In line with the above observations, we found that myocardial I/R reduced the content of phosphorylation endothelia nitric oxide synthase (p-eNOS) (Figures 7B,D) and VE-cadherin in microvascular endothelial cells (Figures 7A,E). This result coincided with the impaired electron-dense endothelial cell (EC)-cell junctions and endothelial barrier integrity (Figures 7C,G) as well as plasma albumin leakage from microvessels (Figures 7F,H). Also, compared to the I/R + Ad-LacZ group, the I/R + Ad-ATF3 group significantly up-regulated the levels of p-eNOS and VE-cadherin on the microvascular surface, improved electron-dense EC junctions and relieved plasma albumin leakage into the myocardium (Figures 7A–H). Collectively, these findings indicate that ATF3 overexpression improves microvascular integrity and permeability in the I/R myocardium.

### ATF3 Overexpression Improved Cardiac Microvascular Perfusion in I/R Injury Rats

**FIGURE 8:**
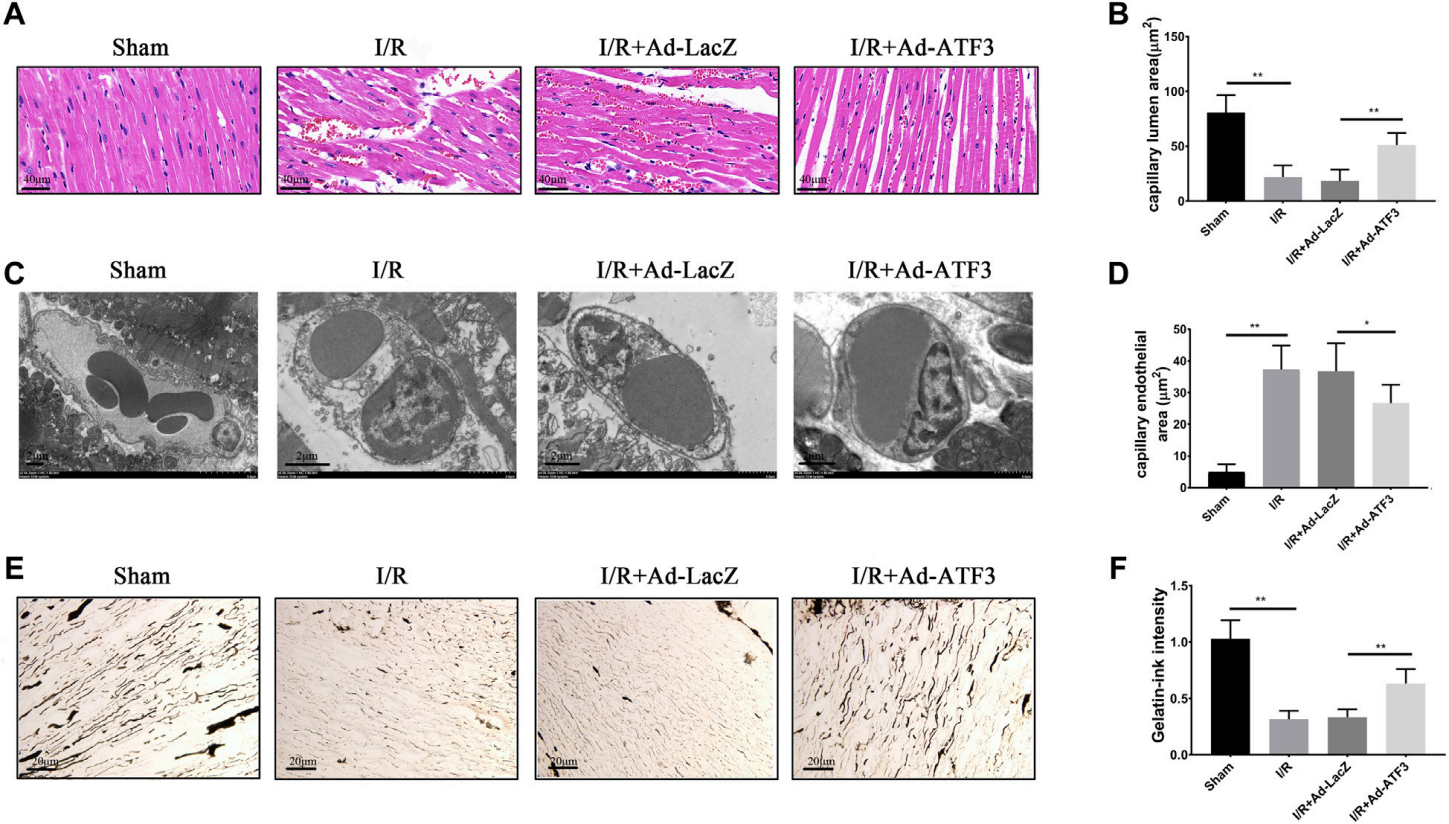
ATF3 overexpression improved cardiac microvascular perfusion in I/R rats **(A)** HE staining shows erythrocyte aggregation and morphological changes following a microvascular blockade **(B, C and D)** TEM was performed to assess the microvascular endothelial and luminal area. I/R-induced EC swelling and hypertrophy, resulting in luminal stenosis, was ameliorated by ATF3 overexpression **(E and F)** The gelatin-ink staining was performed to examine the microvascular perfusion defect. I/R caused the blockage of microvessels which was attenuated by ATF3 overexpression. *p < 0.05, **p < 0.01. n = 8/group.

Since microvascular integrity damage and leukocyte infiltration are the prime causes of microcirculatory dysfunction and myocardial hypoperfusion, we next examined whether ATF3 overexpression plays a role in myocardial tissue hypoperfusion. HE staining showed that the morphology of red blood cells in the myocardium changed into “swollen” or “massed” from “parachute” or “arrow” shapes after I/R (Figure 8A), indicating a microvascular blockade and the interruption of turbulent blood flow. Myocardial I/R also induced endothelial cell swelling, resulting in the narrowing of microvascular lumens. In addition, there was a significant decrease in microvascular luminal area and an increase in endothelial area as analyzed by TEM (Figures 8B–D). Moreover, myocardial I/R induced microcirculation blockage, as revealed by gelatin-ink staining (Figures 8E,F). Compared to the I/R + Ad-LacZ group, the I/R + Ad-ATF3 group significantly ameliorated the altered morphology of red blood cells, relieved endothelial cell swelling, increased the microvascular luminal area and maintained the opening of microvessels (Figures 8A–F). Taken together, these observations support the premise that ATF3 overexpression ameliorates cardiac microvascular perfusion in I/R injury rats.

### ATF3 Overexpression Reduced Infarct Size, Improved Hemodynamic Indices and Heart Function in I/R Rats

**FIGURE 9:**
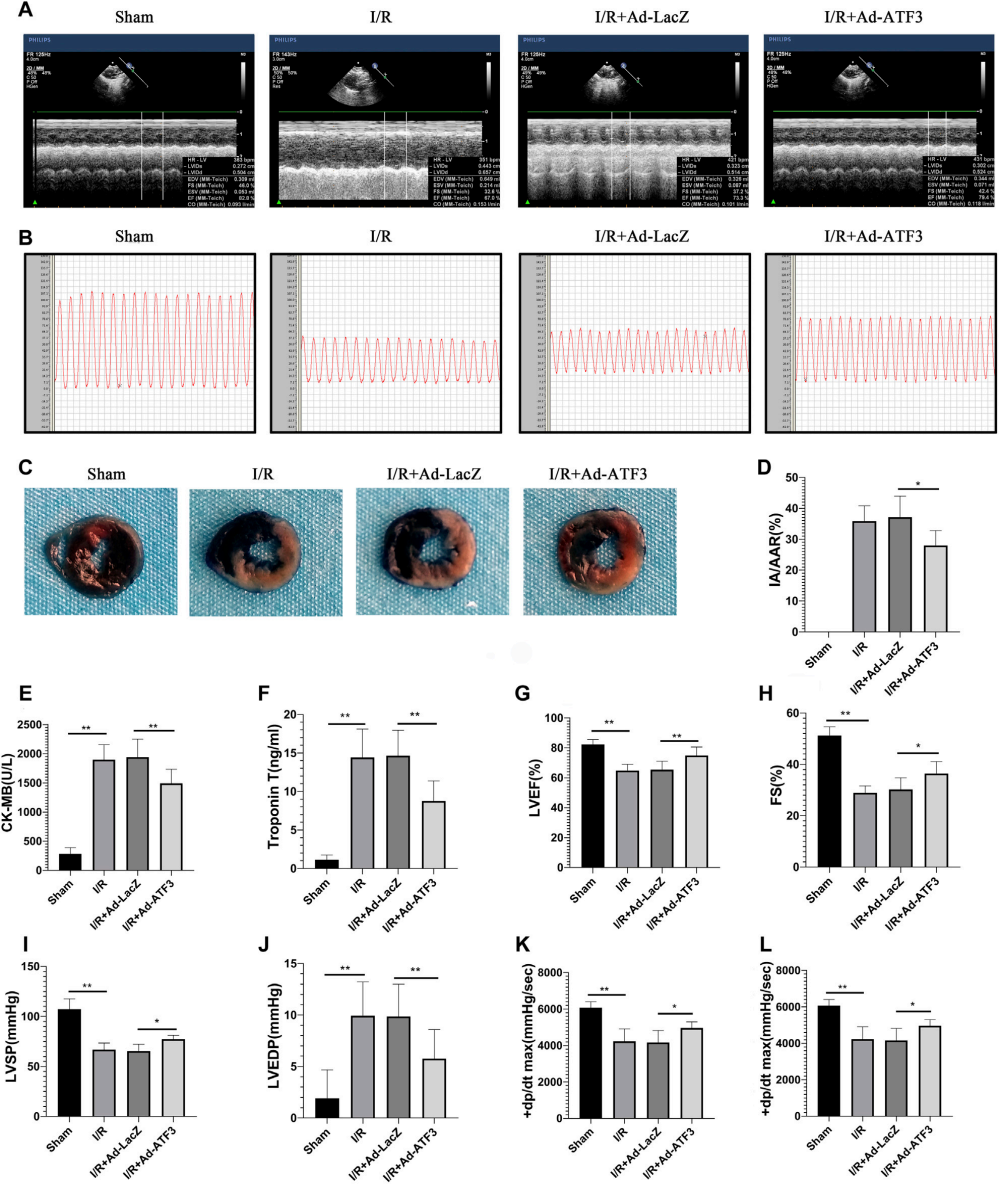
ATF3 overexpression reduces infarct size, improves hemodynamic indices and heart function in I/R injury rats **(A and G, H)** Left ventricular function was determined by echocardiograms **(A)**, including EF **(G)** and FS **(H)** **(B, I-L)** The right carotid artery was intubated to the left ventricle to determine hemodynamic parameters. Representative tracings of LV pressures in each rat are shown in **(B)**. Hemodynamic indices, including LVSP **(I)**, LVEDP **(J)**, and ± dp/dtmax **(K,L)**, were analyzed using the MPA cardiac function system software **(C and D)** Evans Blue and TTC staining were performed to determine infarct size **(C)**. The ratio of myocardial infarct area to area-at-risk (IA/AAR) was calculated **(D) (E and F)** The levels of CK-MB **(E)** and cTnT **(F)** in rat serum were determined using ELISAs. *p < 0.05, **p < 0.01. n = 8/group.

Given that impaired microvascular reperfusion is associated with infarct size and heart function (Nguyen et al., 2015), we examined whether ATF3 overexpression affected infarct area and cardiac function in myocardial I/R rats. The levels of circulating myocardial necrosis marker CK-MB and cTnT were increased following I/R (Figures 9E,F). As expected, I/R injury also increased the infarct area (IA/AAR) (Figures 9C,D) and impaired hemodynamic indices, including a significant elevation in LVEDP and a reduction in LVSP and ± dp/dtmax (Figures 9B,I–L). Moreover, I/R injury impaired cardiac function, as evidenced by a significant reduction in LVFS and EF (Figures 9A,G,H). Compared to the I/R + Ad-LacZ group, the I/R + Ad-ATF3 group exhibited reduced pathological/pathophysiological changes (Figures 9A–L). Thus, ATF3 overexpression reduces infarct size, improves hemodynamic indices and heart function in myocardial I/R rats.

## Discussion

In this study, we investigated the role of ATF3 in I/R-triggered myocardial pathophysiology and the underlying mechanisms. We found that 1) bioinformatics analysis of two GEO datasets related to myocardial I/R showed upregulation of ATF3 in I/R myocardium, 2) ATF3 overexpression inhibited the TLR4/NF-κB pathway activation, inflammation and oxidative stress during H/R, 3) ATF3 overexpression inhibited the TLR4/NF-κB pathway activation, inflammation and oxidative stress during I/R, and 4) ATF3 overexpression inhibited leukocyte infiltration, maintained microvascular integrity and permeability, restored cardiac microvascular perfusion and effectively protected cardiac microvascular ischemia/reperfusion injury, eventually leading to smaller infarct size, improved hemodynamic indices and heart function in myocardial I/R rats.

ATF3 has been shown to be involved in the modulation of a variety of cardiovascular pathophysiologies (Nobori et al., 2002; Kalfon et al., 2017; Zhou et al., 2018b). A recent bioinformatics study on acute myocardial infarction (AMI) demonstrated that ATF3 was a key transcription factor in the immune response to AMI (Zhang et al., 2014). Research also showed that endoplasmic reticulum (ER) stress contributes to ATF3 activation, which in turn mediates the late phase of ischemic preconditioning against I/R injury (Brooks et al., 2014). Consistent with the above findings, bioinformatics analysis of this study also revealed that ATF3 may be involved in regulating I/R progression in rats. Although there are four overlapping genes across the two datasets, the fact that oxidative stress and the TLR4/NF-κB-mediated inflammatory response play an important role in cardiac microvascular I/R injury must be considered. Furthermore, previous studies suggest that ATF3 is associated with TLR4 and oxidative stress. Therefore, in the current work, we selected ATF3 as the subject of interest.

ROS-mediated oxidative stress has long been considered one of the main mechanisms in I/R injury (Yellon and Hausenloy, 2007). When the balance between the generation of ROS and the antioxidant defense systems is interrupted, oxidative stress occurs (Poljsak et al., 2013). In this study, we found that ROS production and the MDA level were upregulated and that the level of SOD and GSH-Px were downregulated in rat hearts subjected to I/R and in H9C2 cells exposed to H/R. Oxidative stresses plays a critical role in the adaptive increase in ATF3 expression (Okamoto et al., 2006; Zhou et al., 2018b; Zhu et al., 2018). However, the mild adaptive up-regulation of ATF3 at reperfusion is not sufficient to protect the myocardium against I/R injury (Yoshida et al., 2008; Koivisto et al., 2014). As is the case for TLR4, ATF3 may also serve as a part of a negative feedback loop to modulate the oxidative stress injury triggered by I/R (Gilchrist et al., 2006). Overexpression of ATF3 further inhibited ROS-mediated oxidative stress, and the underlying mechanism may be related to the up-regulation of the antioxidant enzymes of SOD and GSH-Px (Aung et al., 2016).

In the present study, H/R and I/R activated the TLR4/NF-κB pathway and inflammatory response, as expected. Previous studies also showed that ATF3 is up-regulated by TLR4 activation and act as a part of the negative feedback loop that inhibits the TLR4/NF-κB pathway-mediated inflammatory response (Gilchrist et al., 2006; Whitmore et al., 2007), which is in agreement with observations from the present study. Furthermore, the current results provide evidence that ATF3 overexpression inhibited TLR4/NF-κB pathway activation and down-regulated the expression of downstream proinflammatory cytokines, including TNF-α, IL-1β, IL-6, ICAM-1 and VCAM-1. The inhibition of TLR4/NF-κB signaling provides cardioprotection serves as a promising therapeutic avenue for myocardial I/R injury in cell and animal models (Dong et al., 2018; Yuan et al., 2018). Therefore, we believe that through suppressing the activated TLR4/NK-kB pathway and oxidative stress, ATF3 may be a good candidate molecule for the treatment of myocardial I/R injury.

The microvascular structure of the cardiovascular system consists of single-layered epithelial cells (Zhou et al., 2018d). Compared with cardiomyocyte, microvascular endothelial cells are more exposed to leukocytes present in circulating blood and more vulnerable to I/R injury. Indeed, inflammatory cytokines have been shown to promote the loss of VE-cadherin EC–EC junctions and disrupt the endothelial barrier (Zhou et al., 2018c; Hakanpaa et al., 2018). Our findings from this study are in line with the above observations. TLR4/NF-κB pathway activation up-regulated the expression of downstream proinflammatory cytokine ICAM-1 and VCAM-1, which mediate microvascular endothelial-leukocyte adhesion. Leukocyte adhesion, aggregation, inflammatory cytokines and oxidative stress disrupt the microvascular endothelial barrier via reducing the content of p-eNOS and VE-cadherin in endothelial cells (Zhou et al., 2018c; Zhou et al., 2018e). In the present study, we observed damaged electron-dense EC-cell junctions and endothelial barrier integrity by TEM. Based on this series of mechanisms, inflammatory activation and oxidative stress induce endothelial cell death or decrease endothelial viability, which are the critical downstream events that ultimately contribute to microvascular damage (Zhou et al., 2018a; Cuijpers et al., 2020). This damage eventually causes plasma albumin leakage from microvascular lumen into the myocardium (Zhou et al., 2017a; Zhou et al., 2017b). The interstitial edema may in turn compress the microvascular bed and reduce total cross-sectional vascular area. All these mechanisms contribute to cardiac microvascular I/R injury and microcirculatory perfusion deficit.

**FIGURE 10:**
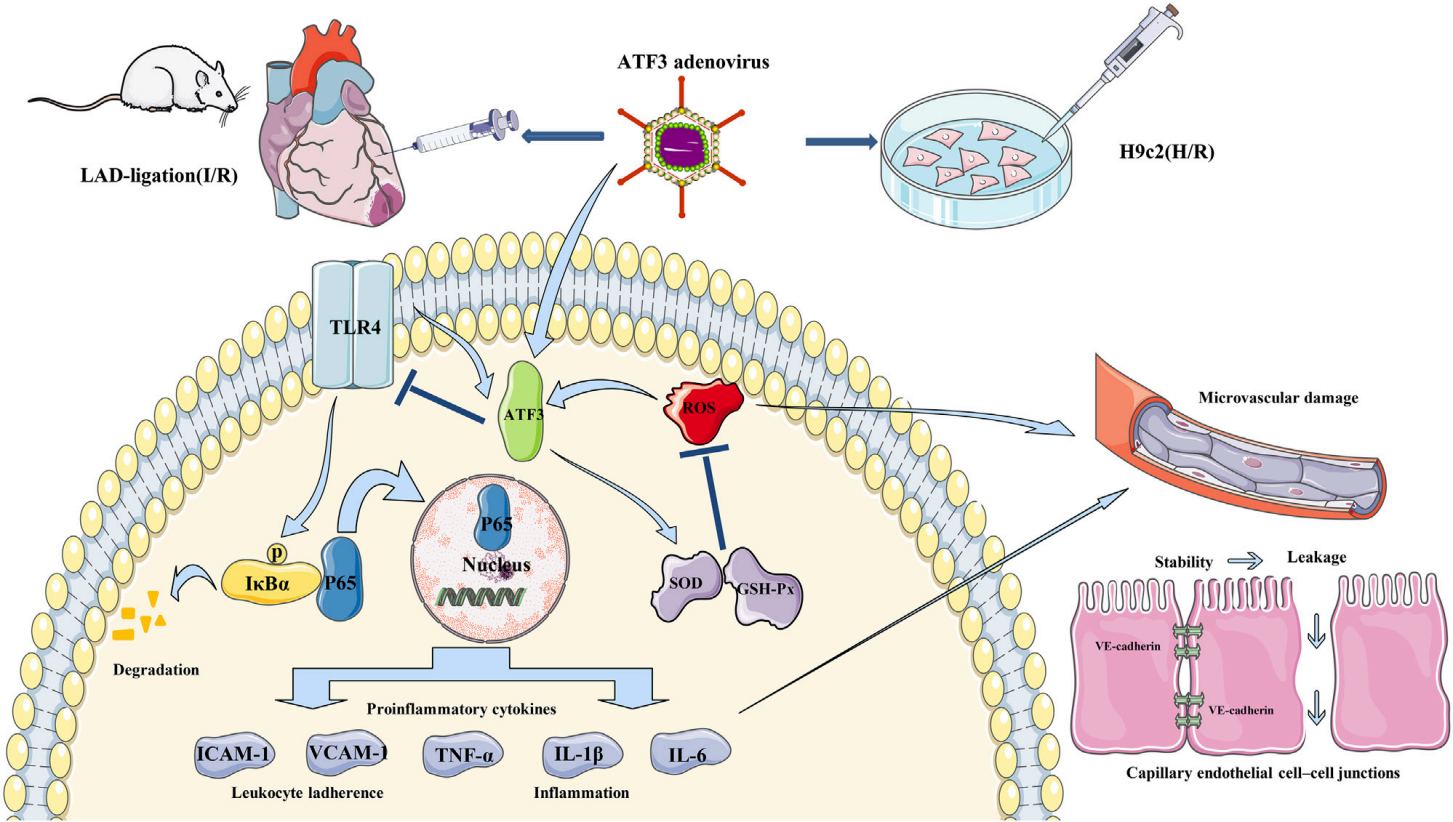
***Schematic showing ATF3 overexpression attenuates cardiac microvascular I/R and H/R injury.*** TLR4 is activated by I/R and H/R, and triggers IκBα phosphorylation and NF-κB p65 nuclear translocation, which subsequently up-regulates the expression of downstream proinflammatory cytokines (TNF-α, IL-1β, IL-6, ICAM-1 and VCAM-1). On the other hand, ROS-mediated oxidative stress occurs in I/R and H/R. These activations disrupt the junctions between ECs by reducing the content of VE-cadherin and increasing permeability. Adenovirus-mediated ATF3 overexpression attenuates I/R or H/R-induced injury by inhibiting the TLR4/NF-κB pathway activation, ameliorating oxidative stress and improving cardiac microvascular I/R injury and cardiac function.

It is well-recognized that adenovirus vectors provide an effective approach for gene transfer (Zhang et al., 2016; Xu et al., 2019). For instance, adenovirus-mediated ATF3 overexpression protected cardiomyocyte from doxorubicin-induced apoptosis at the cellular level (Nobori et al., 2002). In addition, a number of studies confirmed that intramyocardial injection of adenovirus-mediated gene delivery was an effective therapy for myocardial protection (Tenhunen et al., 2006). In the present study, we overexpressed ATF3 in the I/R myocardium through adenovirus-mediated gene delivery. ATF3 overexpression significantly reduced leukocyte infiltration via inhibiting TLR4/NF-κB pathway activation and down-regulating the expression of downstream proinflammatory cytokines (Gilchrist et al., 2006; Whitmore et al., 2007; Boespflug et al., 2014). ATF3 overexpression can inhibit I/R-induced microvascular endothelial dysfunction and endothelial cell injury via the reduction in intracellular ROS generation and increase in SOD activity (Aung et al., 2016). Moreover, ATF3 overexpression improved I/R-damaged electron-dense EC junctions and relieved plasma albumin leakage into the myocardium. Previous findings suggested that ATF3 overexpression prevented the loss of p-eNOS and VE-cadherin in endothelial cells and stabilized microvascular integrity (Hakanpaa et al., 2018; Zhou et al., 2018c). Thus, it is highly likely that ATF3 overexpression attenuates cardiac microvascular I/R injury and improves microvascular perfusion through multiple mechanisms. These mechanisms include improving microvascular integrity disruption and microvascular hyperpermeability which are caused by the TLR4/NF-κB pathway activation-triggered inflammatory response and oxidative stress. Based on this, as shown in Figure 10, ATF3 overexpression attenuates leukocyte infiltration and inflammatory responses by inhibiting the I/R-triggered activation of TLR4/NF-κB pathway signaling. This process in turn alleviates microvascular endothelial cell injury and dysfunction by ameliorating oxidative stress and thus improves the EC–EC junction loss and endothelial barrier disruption. Finally, this cascade of activities eventually benefits the microvasculature by maintaining integrity and permeability, and ameliorating microvascular damage (Aung et al., 2016; Zhou et al., 2017c; Zhou et al., 2018c).

Microvascular perfusion is linked to TnT levels, a large infarct area and LV function post-STEMI (Nguyen et al., 2015). Moreover, the level of microvascular perfusion following primary PCI in STEMI is closely associated with mortality and adverse clinical events (Carrick et al., 2016). Consistent with the above findings, in the present study, we observed I/R-induced cardiac microvascular EC injury and microvascular damage. However, ATF3 overexpression ameliorated microvascular EC injury and microvascular damage, and improved microvascular perfusion to guarantee the delivery of sufficient blood, energy, oxygen and nutrients. This action thus rescued cardiomyocyte health, which eventually decreased the level of troponin T and CK-MB, reduced infarct size and improved cardiac function (Zhou et al., 2018d; Wang et al., 2020). Moreover, cellular injury was observed *in vitro* in a cellular H/R model, and was reversed by ATF3 overexpression. These results also partly explain the change in infarct size *in vivo*. Therefore, ATF3 overexpression eventually improved cardiac function and hemodynamic indices that were compromised by myocardial I/R injury.

There is a major limitation in the present study should be mentioned. Although the animal experiments described here explored the role of ATF3 overexpression in sustaining microvascular structure and function, H9c2 cells were also used as an *in vitro* model in this study. However, the H9c2 cell line is not an endothelial cell line. Therefore, our future studies will use an endothelial cell line to further explore the role of ATF3.

In conclusion, we report in this study that ATF3 overexpression protects the heart against I/R induced myocardial injury and ameliorates cardiac dysfunction. Mechanistically, ATF3 overexpression reduces leukocyte infiltration and inflammatory responses by inhibiting the activation of TLR4/NF-κB pathway signaling triggered by I/R, alleviates microvascular endothelial cell injury by ameliorating oxidative stress, maintains microvascular integrity and permeability, and restores cardiac microvascular perfusion. Preventing microcirculatory reperfusion damage will be a major focus in future treatment strategies. The present study suggests that the ATF3 overexpression may be a potential molecular-targeted therapy for microvascular reperfusion injury and no-reflow.

## Data Availability

The raw data supporting the conclusions of this article will be made available by the authors without undue reservation to any qualified researcher.
